# Usability of an exosuit in domestic and community environments

**DOI:** 10.1186/s12984-022-01103-6

**Published:** 2022-12-01

**Authors:** Chiara Basla, Irina Hungerbühler, Jan Thomas Meyer, Peter Wolf, Robert Riener, Michele Xiloyannis

**Affiliations:** 1 Sensory-Motor Systems (SMS) Lab, Institute of Robotics and Intelligent Systems (IRIS), ETH Zürich, Zürich, Switzerland; 2 Spinal Cord Injury Center, Balgrist University Hospital, Medical Faculty, University of Zürich, Zürich, Switzerland; 3 Rehabilitation Engineering Lab, Institute of Robotics and Intelligent Systems (IRIS), ETH Zürich, Zürich, Switzerland

**Keywords:** Exosuits, End-user perspective, Technology acceptance, Daily life settings

## Abstract

**Background:**

Exosuits have been shown to reduce metabolic cost of walking and to increase gait performance when used in clinical environment. Currently, these devices are transitioning to private use to facilitate independent training at home and in the community. However, their acceptance in unsupervised settings remains unclear. Therefore, the aim of this study was to investigate end-user perspectives and the adoption of an exosuit in domestic and community settings.

**Methods:**

We conducted a mixed-method study to investigate the usability and user experience of an exosuit, the Myosuit. We leveraged on a cohort of seven expert users, who had the device available at home for at least 28 days. Each participant completed two standardized questionnaires (SUS and QUEST) and one personalized, custom questionnaire. Furthermore, a semi-structured interview with each participant was recorded, verbatim transcribed and analyzed using descriptive thematic analysis. Data collected from device sensors quantified the frequency of use.

**Results:**

A mean SUS score of 75.4 out of 100 was reported. Five participants scored above the threshold for above-average usability. Participants also expressed high satisfaction with most of the technical features in the QUEST with an average score of 4.1 (3.86–4.71) out of 5. Participants used the Myosuit mainly for walking outside and exercising at home. However, the frequency of use did not meet the recommendations for physical activity established by the World Health Organization. Five participants used the Myosuit approximately once per week. The two other participants integrated the device in their daily life and used the Myosuit to a greater extent (approx. five times per week). Major factors that prevented an extensive use of the technology were: (i) difficulties in donning that led to (ii) lack of independence and (iii) lack of motivation in exercising.

**Conclusions:**

Although usable for various activities and well perceived, the adoption of the exosuit in domestic and community settings is yet limited. Use outside the clinic poses further challenges that should be considered when developing new wearable robots. Primarily, design should meet the users' claim for independence and increased adjustability of the device.

**Supplementary Information:**

The online version contains supplementary material available at 10.1186/s12984-022-01103-6.

## Introduction

Many different pathologies, such as multiple sclerosis, muscular dystrophy, and spinal cord injury, lead to lower limb impairment and restrict people’s ability to perform physical activity. Most people suffering from lower limb impairments are physically inactive [1–3], failing to fulfil the World Health Organization (WHO) recommendations of at least 150 to 300 min of moderate-intensity aerobic physical activity per week [4]. As a consequence of a sedentary lifestyle, physical deconditioning results in severe secondary conditions [5] that markedly impact the quality of life [6, 7]. Additionally, reduced mobility hampers the ability to accomplish activities of daily living [8]. For all these reasons, having the possibility to perform physical activity and stay active after being discharged from hospital or during pathology progression is extremely important. In this context, exosuits have the potential to enhance the intensity and the dose of gait training across the continuum of care, to achieve personal mobility at home and in the community [9].

The potential of exosuits to support walking has been demonstrated by reduced energy expenditure during walking [10–15] and by improved gait speed, walking distances and ambulation ability [16–19]. Despite the increasing number of studies investigating functional improvements of participants using robotic devices, little research has focused on end-user perspectives in term of usability and user experience [20]. Manns et al. reported participants’ positive experiences about learning to walk with the ReWalk during and after 4 times weekly training sessions over 12 weeks [21]. Poritz et al. used a customized questionnaire and free response questions to compare user satisfaction during REX and Ekso exoskeleton-assisted training after a minimum of 5 training sessions with each device [22]. Other studies reported first-impression usability evaluations, after a 1.5 h training session with the H2 exoskeleton [23] or after showcasing videos of the ReWalk, the Ekso and the Rex to 13 incomplete spinal cord injured participants [24]. These studies were all performed with rigid exoskeletons within clinical or laboratory environments. However, investigating end-user perspectives in-the-lab or in clinical settings might not reflect context-specific factors that rise from at-home use of exoskeletons and that influence adoption of technology in unsupervised environments [25]. For this reason, investigating exoskeleton use at home and in the community has been often highlighted as a core paucity of the literature, although such investigations would inform the design and development process of wearable robots [22], as well as are expected to facilitate exoskeletons’ uptake in uncontrolled environments and to reduce the abandonment rate [26]. The study from Van Dijsseldonk et al. was the only one to assess where, for how long and for what purpose people used the ReWalk exoskeleton at home and in the community during a 2 to 3 weeks period. The study demonstrated extensive use of the device, especially for exercises purposes and social interaction but low potential for activities of daily living. Weight, ease of use and safety were indicated as main limitations of the device [27].

The level of support provided by an exosuit is limited compared to that of rigid exoskeletons, and the targeted users typically present greater residual capability. Therefore, we expect their experiences and expectations to be different from users with complete spinal cord injury. To the best of our knowledge, no studies discussed the purpose for which exosuits were used nor their usability and user experience in daily settings. These insights would allow for an in-depth understanding of wearable robots’ acceptance in domestic and community settings.

The aim of this study was to (1) evaluate the daily life usability of an exosuit, (2) investigate how the device is used in domestic and community environments and (3) highlight the factors that facilitate and limit the acceptance and adoption of exosuits in unsupervised settings. This study leveraged on a unique group of expert users that could extensively use a exosuit in different scenarios of daily living, both in domestic and community settings. For addressing our research questions, we investigated end-user prospective on the Myosuit (MyoSwiss AG, Zürich, Switzerland), a commercially available exosuit that supports walking, stair climbing, and sit-to-stand transfers. The Myosuit was originally designed to be used as a training device in clinical settings but was subsequently transitioned to private use to allow users to continue exercising independently at home and in the community.

Importantly, our study is not a clinical study to test the efficacy of an exosuit but a case series on its effectiveness in real-world scenarios. Compared to efficacy study, effectiveness studies examine an intervention under more realistic circumstances that better resemble real-world scenarios, with heterogeneous sample population and less standardized protocols [28]. This case series serves as a blueprint to inform developers and researchers on the essential requirements that an exosuit should meet to be well accepted within the community and extensively used in unsupervised settings. Furthermore, the study paves the way for a longitudinal study investigating whether independent use of wearable robots at home increases mobility and activity level, allowing limited household and limited community walkers to reach the minimal physical activity threshold required for health benefits.

## Methods

### Participants

Seven volunteers participated in the study (demographic data provided in Table 1). To be eligible for participation in this study, the following inclusion criteria were established: (1) having had the Myosuit at home for at least four weeks; (2) confirmed diagnosis of pathology leading to muscular weakness of the legs; (3) being over 18 years of age; (4) no other diagnosis that has prevented the use of the device in the four weeks prior recruitment; (5) being able to understand the informed consent form, the written questionnaires, and the verbal open-ended questions. The authors contacted the company commercializing the device (MyoSwiss AG, Zürich, Switzerland) and received a list of their customers (users). The users were approached by email and those who agreed to participate returned the informed consent form signed. This study was approved by the ETH Zurich Ethics Committee under application number 2021-N-56.

**Table 1:** Participants’ demographics

Participant	Age	Gender	Pathology	Time since first diagnosis (years)	LEFS score
P1	49	F	Multiple Sclerosis	8	19
P2	54	M	Spinal Muscle Atrophy	24	17
P3	56	M	Spastic Paresis	N/A	23
P4	49	M	Bethlem Myopathy	6	33
P5	52	F	Multiple Sclerosis	9	32
P6	56	M	Multiple Sclerosis	24	22
P7	34	M	Cauda-equina syndrome	10	46

*LEFS* Lower Extremity Functional Scale, a self-administered questionnaire that evaluates the functional ability of participants to perform activities of daily living while not assisted by any assistive device [29]. Higher LEFS score indicates higher functional ability (maximum score = 80)

### Wearable robot

**Fig. 1:**
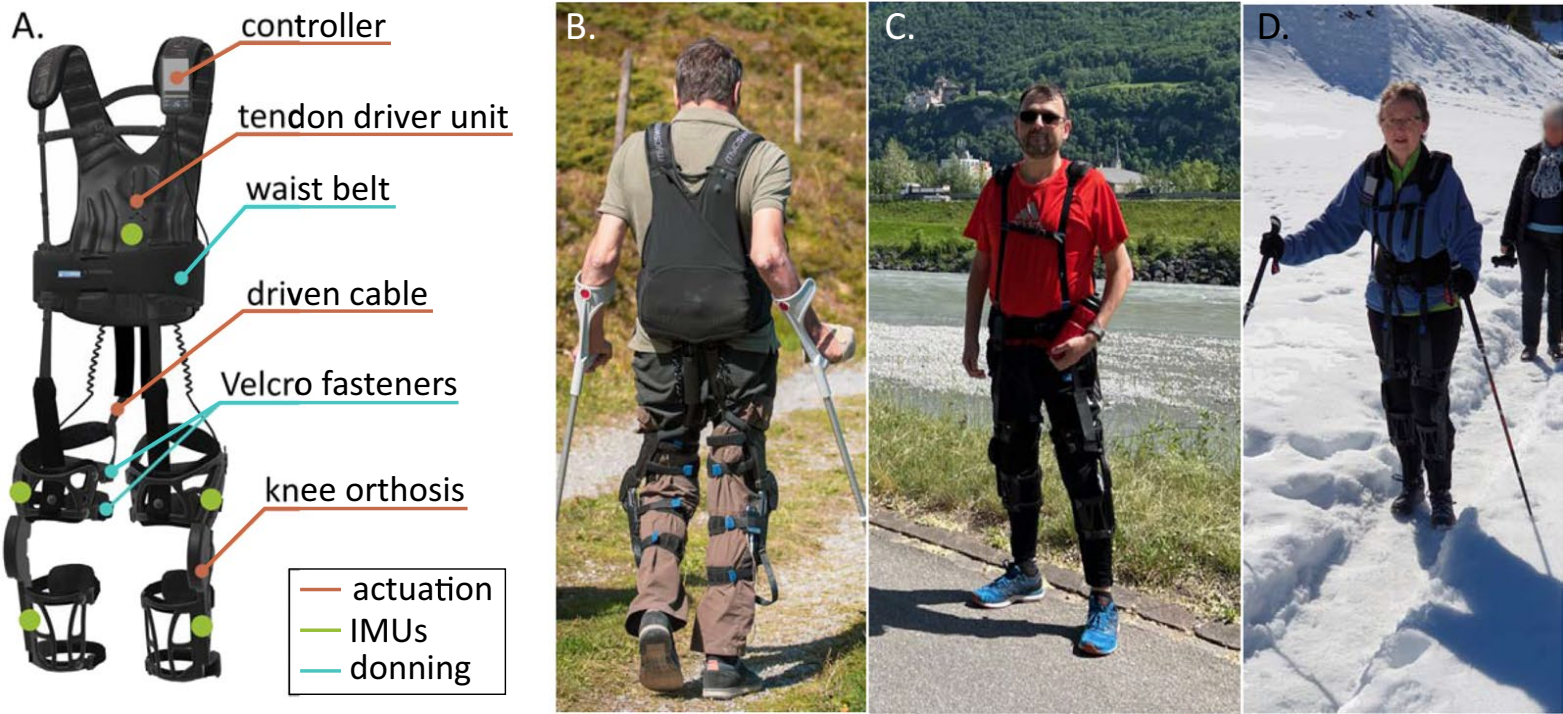
The Myosuit. **A** Main components of the exosuit used in the study are highlighted. The Myosuit is a cable-driven lightweight wearable robot. Motors in the tendon driver unit apply an extension torque at the hip and knee when pulling the cables routed along the thighs. Inertial measurement unit (IMU) sensors determine the posture and segment the gait to adapt assistance to the user’s movements. **B**–**D** Myosuit’s users wearing the Myosuit in the community and performing activities of daily living with the device

The Myosuit (Myosuit Gamma, MyoSwiss AG, Zürich, Switzerland) was used for the purpose of this study (see Fig. 1). The Myosuit is a compact and lightweight wearable robot designed to support people with lower limb motor impairments in performing walking, sit-to-stand transfers and stair climbing. The wearable robot consists of a motor tendon driver unit to be worn at the back, containing all the electronics and two motors, and two knee orthoses. One cable is routed from each motor, over the buttocks, around the thigh and fixed at the shank. When the cable is retracted both the hip and the knee articulations are simultaneously extended. Forces are applied during the stance phase of the gait, when the leg is bearing the weight of the body, and inertial measurements units (IMU) embedded in the suit are used to detect gait events and provide assistance when needed [30]. The Myosuit weights 5.6 kg (including the battery) and approximately 75% of the weight is concentrated in the backpack, close to the user’s center of mass. The device is tightened and adjusted to the user’s body using a textile wait belt and four Velcro fasteners on each leg. A video showing the donning process and additional information on the device can be found on the company webpage [31].

### Study design

The study was designed as a mixed-method research in which quantitative and qualitative data were collected through questionnaires and a semi-structured interview, respectively.

Each participant filled in two standardized questionnaires. First, the Quebec User Evaluation of Satisfaction with Assistive Technology 2.0 (QUEST) questionnaire measured the users’ satisfaction with the wearable technology. Participants rated on a five-point Likert scale (from “not satisfied at all” to “very satisfied”) their satisfaction in terms of dimensions, weight, adjustments, safety, durability, simplicity of use, comfort, and effectiveness. The scores were converted into a scale from 1 to 5 to compute the average. Second, the System Usability Survey (SUS) evaluated the device usability with ten statements on device usability to be ranked on a five-point Likert scale (from “strongly disagree” to “strongly agree”). A SUS score from 0 to 100 was obtained for each participant as explained in [32]. Additionally, we designed a personalized questionnaire to evaluate the location and purpose of use, device fitting, independent use, learnability, ease of control and acceptance within the community (see Additional file 1: Table S1). The personalized questionnaire was developed based on previously published surveys [22, 33] and the clarity of the questions’ phrasing was tested with three members of our research group not involved in the preparation of the questionnaire. Demographic data (age, sex, living environment, etc.), and diagnosed pathologies were collected for each participant at the end of the personalized questionnaire. Finally, participants took part in a semi-structured interview of approximately one hour with open-ended questions to let participants express more freely opinions and concerns regarding their personal experience with the Myosuit. The guideline of the interview (see Additional file 1: Table S2) was developed based on previously published guides [23, 34, 35]. Participants were instructed to answer as honestly as possible to all the written and oral questions.

Additionally, we gathered inertial data automatically collected from five IMU sensors embedded in the Myosuit (one in the backpack, one on each thigh and one on each shank, see Fig. 1), and we extracted metrics describing the activity level of the user, namely number of sessions per month, number of steps per session and duration of each session. One session was considered as any time the device was turned on and used for at least five consecutive minutes. We accessed data from the day participants first had the device at home until the day of the interview. These metrics quantified the amount of time the device is used by each participant and were integrated and compared to the questionnaires and interview answers to draw a more exhaustive description of the device usage.

Quantitative data from the questionnaires was collected in Microsoft Excel (Microsoft Corporation, US). Questionnaires and inertial data were analyzed using Matlab (MATLAB R2021a, The MathWorks, Inc.). Being a case series, we did not perform any statistical analysis on the data, and we presented individual results for each participant.

### Qualitative data analysis

All questionnaires and interviews were held in the native language of the participant by investigators (IH, researcher and FM, researcher in Acknowledgement) that fluently spoke the language. All participants were native German speakers. Each interview was audio-recorded, verbatim transcribed and translated to English (IH and FM translated the interviews; CB (doctoral researcher) verified the accuracy of each translation before the analysis). Descriptive thematic analysis [36] was conducted to analyze the interviews’ transcripts. An inductive approach was used when analyzing the transcripts [37], meaning that broad comprehensive themes were gathered from the collected data. Specifically, the main ideas of each transcript were coded and the data that appeared to be comparable between participants segmented to extract the more recurrent topics. This process required an iterative approach, continuously revising the themes as soon new data were collected and new codes generated. NVivo software (version 1.5.1, QRS International, Australia) was used to support data analysis. Thematic analysis was stopped when no new themes emerged. Independent parallel coding was applied to assess coding consistency. Two investigators (CB and IH) read and coded the transcripts separately. For each transcript, the investigators met to discuss the identified codes and evaluate interpretations. Once all transcripts were individually analyzed, each researcher developed specific themes and subthemes. Subsequently, the identified codes, subthemes and themes were merged, and further discussed whenever overlap between investigators’ findings was low. The final definition of the themes was discussed with the main investigator (MX, postdoctoral researcher).

To ensure accuracy and trustworthiness of the findings, participants were recontacted after their transcripts were analyzed [34]. Each participant revised a document presenting the synthesized analyzed data of his/her transcript to guarantee that the findings matched his/her experience. Any changes or added information was integrated into the final analysis.

## Results

### Usability

**Fig. 2:**
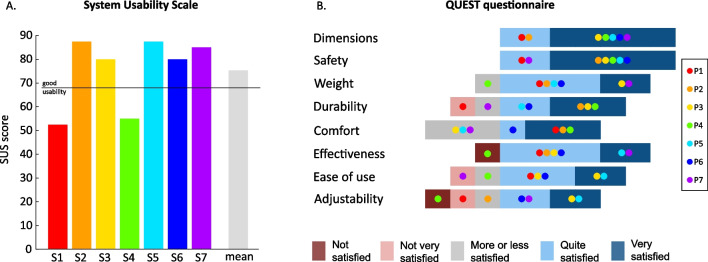
Usability questionnaires. **A** System Usability Scale. SUS scores for each participant and mean across participants show usability of the Myosuit above average. Each colour represents one participant. The grey bar represents the mean across participants. **B** Quebec User Evaluation of Satisfaction with assistive Technology. High satisfaction with most of the technical features of the Myosuit is shown. Individual results for each participant are reported using different colours

The SUS score for each participant ranged from 52.5 to 87.5 (see Fig. 2A) and the average score was 75.4. In the QUEST questionnaire, satisfaction with each technical feature of the device ranged from 3.86 to 4.71, with an average score across all participants and all features of 4.1 (see Fig. 2B). Ease of transport, satisfaction with donning/doffing and satisfaction with the level of gained independence obtained the lowest median scores in the personalized questionnaire (see Additional file 1: Fig. S1).

### Performed activities

**Fig. 3:**
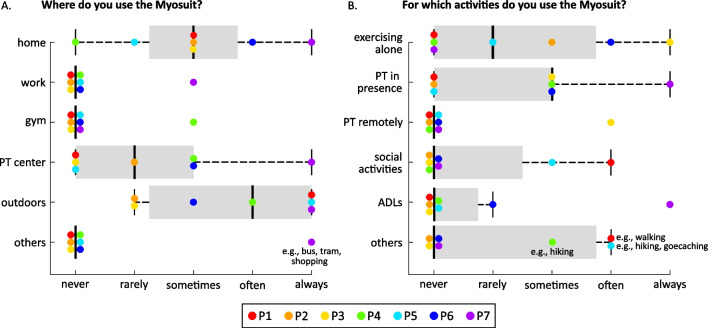
Location and purpose of use of the Myosuit. Subjects were asked to report how often they used the Myosuit **A** in different locations and **B** for different activities. The Myosuit were mostly used outdoors for walking either alone or with a physiotherapist. Most of the participants frequently used the Myosuit at home for exercising alone or with a physiotherapist. Only one participant uses the Myosuit frequently for activities of daily living. Each colour represents one participant. The thick black line indicates the median, and the bottom and top edges of the box indicate the 25th and 75th percentiles, respectively. The whiskers extend to the most extreme data points not considered outliers. *PT* physiotherapist, *ADLs* activities of daily living

Three out of the seven participants reported to use the Myosuit mainly outdoors, while three participants used the Myosuit almost solely inside their houses (see Fig. 3A). Only one participant reported to frequently use the Myosuit both outdoors and at home, as well as at a physiotherapy center. None of the participant regularly used the Myosuit at work or at a gym.

Inside the house, the Myosuit was exclusively used for exercising, either alone or with a physiotherapist remotely (see Fig. 3B). Outdoors the Myosuit was mainly used for exercising, either alone or with a physiotherapist. Three participants used the Myosuit outdoors for leisure activities (e.g., walking) with a family member, for social activities in the community (e.g., geocaching) or for activities of daily living (e.g., shopping).

### Frequency and time of use

IMU data from participant P2 were not properly recorded due to technical issues of the device and hence could not be used for further analysis. Four out of the six participants for which data were available used the device on average approximately once per week (see Table 2). Greater use of the device was recorded for two participants, which used the device on average approximately five times per week. We did not find a notable difference on the average duration of the sessions between participants (median of 46.1 min). Although session’s duration was similar, participant performed considerably different number of steps (range from 252.1 to 4256.7 steps).

**Table 2:** Participants’ activity level

	P1	P3	P4	P5	P6	P7
Total weeks of use	9.2	18.0	20.0	8.0	8.4	11.6
Total number of sessions	9	19	18	37	9	60
Mean number of sessions per week	1.0	1.0	0.9	4.6	1.1	5.2
Mean number of steps for session	2125.3	252.1	4256.7	2244.5	681.4	3457.5
Mean session duration (min)	61.5	35.5	45.1	66.7	47.0	44.1

Participants’ activity level was tracked with inertial measurement units embedded in the device

### Qualitative analysis

**Fig. 4:**
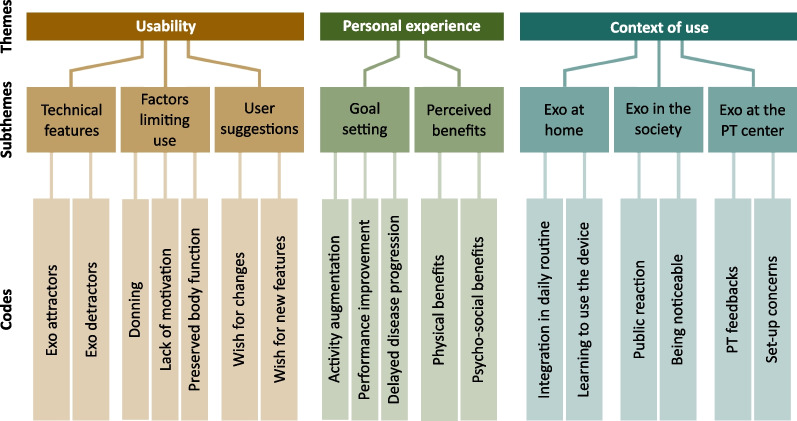
Thematic analysis results. Three themes were identified during thematic analysis: (1) Usability; (2) Personal experience; and (3) Context of use. Each theme is divided into subthemes and codes

Three themes were identified during thematic analysis: (1) usability; (2) personal experience; and (3) context of use. Within each theme, codes were grouped, and multiple subthemes were identified (see Fig. 4). In this section we report exemplary statements for each theme, taken directly from the interview transcripts, using the wording from the participants (additional quotes in Additional file 1: Table S3).

#### Usability

This theme discusses the usability of the Myosuit in terms of (i) satisfaction towards the technical features of the device and (ii) factors that limit its use in daily life settings. Furthermore, (iii) user suggestions on how to improve the usability of the device were included within this theme.

All participants considered the Myosuit compact and were satisfied with its dimension and weight. The backpack was not perceived as heavy while walking but rather disturbing when sitting for a prolonged time and using the car. Similarly, comfort at the interface was maintained during walking but was mentioned as problematic during long sitting periods.

“The backpack is not perceived as disturbing weight. Once you have it on and turn the Myosuit on, that's not anymore. So, from the outside, it may seem that it's way too heavy, but this is not the case at all.” (Participant P2)

“[The straps of the Myosuit] tie you up pretty much when you have to sit somewhere longer.” (Participant P1)

The Myosuit was described as easy to control and all participants could operate it independently.

“There [operating the device] I'm mostly independent. I can make the settings alone.” (P7)

Users were satisfied with the support provided by the Myosuit, especially in term of increased safety and confidence while walking. However, since the robot does not support hip and knee flexion, participants struggled performing some activities of daily living, such as stairs climbing, as they could not independently reach enough foot clearance. In this regard, support to additional joint movements was desired. Participants expressed disappointment with the fasteners around the thigh and the shank.

“I don't really like Velcro fasteners for myself personally. They get trapped everywhere, right?” (Participant P4)

The donning process was mentioned by most of the participants as the main limitation of the Myosuit. Most participants could either not go through it independently or the time required for it was too long. The three participants that considered themselves satisfied with the donning process could do so in less than 5 min, compared to the other users that took up to 15 min.

“It's hard for me to be able to use the Myosuit on a regular basis. Because I always depend on someone. And that's frustrating in a certain way. If I put on the Myosuit on myself and don't need someone for it, then I would go walking [alone] with the walker too.” (Participant P1)

Limited time availability and exhaustion after a working day were also broadened up as explanations for the limited frequency of training with the Myosuit. Additionally, preserved abilities to ambulate inside the household without any assistive device limited use of the Myosuit during activities of daily living at home.

“I'm still working 100%. For me, normal everyday life is already extremely exhausting. It's practically a matter of time, too” (P4)

“At home in the house I don't use it. I just try the stuff I can still do without [the Myosuit]. My legs are still enough at home.” (P5)

User suggestions on how to improve the usability of the Myosuit were collected during the study. The most frequent answers related to the wish for a simpler design in terms of easier-to-don exosuit, and new features such as a touch screen controller or the possibility to control the device using a smartphone or a smartwatch. Some participants described very futuristic designs.

“The ideal device would be normal pants with the technology inside.” (Participant P2)

#### Personal experience

This theme describes the aspects that motivated participants to use the Myosuit while having it at their disposal at home. Particularly, we discussed (i) the goals set by the users and (ii) the perceived benefits.

Three main goals set by the users were identified. Firstly, participants that primarily used the Myosuit outdoors aimed at improving their walking abilities in terms of walking speed and distance covered. This would allow them to perform once again activities that were prevented by their lower-limb weakness. Secondly, participants used the Myosuit to improve their walking pattern and, thirdly, to delay the progression of their disease.

“My goal is to walk longer distances so I can go hiking in the mountains again.” (Participant P5)

“The goal is simply to improve the gait pattern so that I walk easier and better.” (Participant P3)

“I want to try to keep this walking ability, but also the strength in the legs. Because I'm going worse and worse all the time and if this "worsening" is slowed down, then I'm benefiting from it.” (Participant P6)

Participants were also motivated to continue using the Myosuit by the positive benefits and results that they experienced. We distinguished between physical and psycho-social benefits. Furthermore, we divided physical benefits into restorative (or therapeutic) and assistive benefits depending on whether they were mentioned to persist after removing the Myosuit or not, respectively. In fact, all participants explicitly expressed their excitement towards preserved improvements for a couple of days after a robotic-assisted training.

Restorative benefits included reduced tiredness and fatigue, improved walking pattern, greater stability, reduced pain and increased strength and endurance. One participant mentioned improved bladder function and, consequently, improved quality of sleep.

“I was almost not able to carry a cup of tea from the kitchen to the dining table at first. I spilled the tea because I wasn't stable enough from the pelvis and even jerked in the movements. And now I can transfer a cup of tea on my own [without the Myosuit], simply because the stability of the pelvis improved.” (Participant P1)

“It prevents me from getting pain in joints that are loaded incorrectly or also muscles.” (Participant P3)

Assistive effects included the possibility to walk longer and faster, reduced need to concentrate on the walking task and increased safety.

“[Without the Myosuit] I am very much slower when I move. With the Myosuit I'm always quicker at the destination.” (Participant P7)

“I sometimes block out my surroundings because I'm just concentrated on walking. And with the Myosuit is usually not as strong as without.” (Participant P6)

Most participants did not experience any social benefits. Two participants mentioned increased family interaction and participation in the community. Participants perceived psychological and emotional benefits in terms of increased life satisfaction and, in general, positivity connected to the possibility of being more active.

“I did not notice improvements within the society. I have such a good environment.” (Participant P2)

“Social is going again in the wood with my husband and moving outside with others clearly.” (Participant P5)

#### Context of use

This theme discusses how context of use influenced user experiences with the Myosuit. Specifically, we considered (i) home, (ii) physiotherapy center and (iii) the community as physical environments to which an exosuit could be exposed.

No need to commute and the possibility to integrate the training in the daily schedule at will (without ahead planning) were mentioned as factors that increased the frequency and time of training. However, the latter advantage vanished if assistance from another person was needed to don the device.

“You can individually incorporate it into your day. It has the advantage that I can train more often then, not just once a week. You can use Myosuit when it's convenient.” (Participant P3)

“I don't think it's an advantage now that I have it at home. Because if I don't automatically have someone to help me. It takes a lot more organization as if I have a fixed appointment.” (Participant P1)

In this regard, being at a physiotherapy center was considered an advantage, together with the possibility to receive expert’s feedbacks and corrections.

“I appreciate doing it with therapeutic support because I have to concentrate so much on the movements, that I often don't notice if I'm moving in the wrong way. And with a therapist, they can correct you. Which is also motivating, of course.” (Participant P6)

All participants highlighted the importance of being properly introduced to the device to use it efficiently and independently at home. Being correctly introduced to the Myosuit was extremely important not only for the user but for the physiotherapist too. Participants experienced situations in which the physiotherapist was either unable to deal with the device or preferred to stick to conventional therapy.

“He didn’t know what exercises he was supposed to do with me because he doesn't have the experience with devices like that, so he asked me to train without it.” (Participants P2)

Finally, the community showed a positive attitude towards the Myosuit in many circumstances. Participants reported interest and curiosity in the community, which could become annoying in case of excessive questioning. With respect to this, being conspicuous in the community was discussed but did not represent a problem for any of the users, apart from the same one participant that preferred to hide in the society not to receive questions.

“When walking around, then of course one is a certain eye-catcher, in a way. But that's totally fine for me. Because also with my wheelchair, even when I'm walking, I am always visible.” (Participant P6)

“Then of course everyone comes and wants to know how it works. You can't go anywhere without being addressed. And that takes longer. It's also really tedious for my family.” (Participant P4)

## Discussion

The Myosuit was designed as a training device to be used in clinical settings. Recently, private individuals have also been able to purchase the device to use it themselves at home and in the community at any time. The study aimed at surveying these first private buyers regarding the usability of the device and their experiences in daily life.

### Daily life usability of the Myosuit was scored above-average in standardized questionnaires

Standardized questionnaires showed good usability. Five out of seven participants scored in the SUS questionnaire above the above-average usability threshold set to 68 [38]. In the same questionnaire, all the participants scored above 50 points which is considered the lower bound for device acceptability [39]. However, SUS scores between 70 and 80 were reported in the literature not to guarantee high acceptability of the device [39]. The QUEST questionnaire showed high satisfaction with all the technical features considered. Users felt safe and the lightweight design of the Myosuit was effective, both in terms of dimensions and weight. Marginal usability of the Myosuit and low satisfaction with device’s adjustability matched the inability to don the device independently and the need to always rely on a companion for fitting. We speculate that this aspect also influenced the low score for ease-of-use, since all participants considered themselves satisfied with operating and controlling the device and could do so without external support.

We demonstrated similar usability but higher user satisfaction compared to a rigid exoskeleton [27]. While participants using a rigid exoskeleton were mainly dissatisfied with its weight, effectiveness and safety, all participants of our study, apart from one, considered themselves satisfied with these technical features (QUEST item score ≥ 4). Surprisingly, most participants using a rigid exoskeleton were satisfied with its adjustability, contrarily to the participants of our study that scored this feature the lowest. We believe that this difference relates to the fact that donning of the rigid exoskeleton had to be performed by a companion and participants in [27] did not take the possibility of independent donning into account.

### The exosuit was used in various scenarios of daily living, but with limited frequency

Participants used the Myosuit in various scenarios of daily living, mainly walking outside and exercising at home. Our findings regarding location and purpose of use are similar to those reported in [27]. Both rigid exoskeleton and exosuits were mainly used outdoors for exercising. Our study showed that an exosuit can additionally be used for exercises at home. Since none of the participants trained at home without the Myosuit, exercising at home with an exosuit can be considered as additional training that would not be possible without having the device at disposal at home. Consequently, we can speculate on increased mobility connected to independent use of the exosuit in domestic settings. However, none of the participants used the Myosuit as an assistive device inside their house. During the interviews, participants expressed a clear preference towards simple non-actuated orthosis or solving tasks with their residual abilities. This finding relates to the significantly smaller level of impairment that affects exosuit’s users compared to complete SCI patients [40], and should be kept in mind when considering the context of use and the target population for newly developed exosuits.

Our findings differ from previous published results in terms of exoskeleton’s frequency of use. While we showed frequency of use of approximately once a week for the majority of participants, Wrigth et al. reported a mean home-use of the AlterG Bionic Leg orthosis for training purposes of more than 5 times per week over 10 weeks [41]. Van Dijsseldonk et al. reported a median ReWalk use of 9 days over 2 weeks [27]. Since patients were given a robot for the purpose of the study, the authors themselves speculated that an investigator bias might have influenced the results and encouraged a more frequent use of the device. Additionally, both studies extracted the total number of sessions from physical activity diary and logbook filled in manually by the participants. On the contrary, taking part to our study did not affect the time of use of the device since all data were collected retrospectively and directly from the device on-board sensors.

### Independent donning and use are required to boost training and a more regular use in activities of daily living

Combined analysis of the qualitative and quantitative results allowed us to elaborate on the factors that prevented an extensive use of the Myosuit in unsupervised settings. The two participants (P1 and P4) who reported not being able to wear the Myosuit independently scored the lowest both in the SUS and in the adjustability attribute of the QUEST and their frequency of use was limited to once per week. Limited use was also recorded for the participants (P2 and P6) who stated taking too long for proper independent donning. On the contrary, participants (P5 and P7) who did not mentioned donning as a constrain, used the device more extensively, up to five times per week. Donning was therefore considered the biggest limiting factor and independence in donning remarkably affected the frequency of use. Donning should be fast, performed independently, and easy to carry out (i.e., not tiring). Although the device can be easily dressed on a patient by a physiotherapist or an experienced companion in approximately 2 min, this time was mentioned to increase up to 15 min when a user tries to don the device independently. Considering that sessions lasted, on average, 50 min, the donning process increased the total session duration by 30%, when performed independently. In agreement to [42], fitting time should be reduced to five minutes. Independent use was described to play a fundamental role in the acceptance of the device. Additionally, independent donning was mentioned to facilitate more regular training sessions, not having to accommodate the needs of the companion, and consequently increase the frequency of use. Future development efforts should clearly envision the level of supervision that is expected for their device and adjust the design accordingly, since unsupervised, independent use brings extra challenges that inherently limit the device’s usability [20]. The design of a physical human–robot interface should consider users’ weakness in the lower limbs, movement limitations, reduced stability, and poor handling capabilities. Reduced donning and doffing time, independence in the fitting process and increased comfort during prolonged use are the core requirements for the development of an innovative body attachment system. In this direction, users mentioned the willingness of getting rid of Velcro fasteners and of tightening mechanisms that do not get trapped into other clothes, such as magnetic clips. An efficient way to transfer forces to the human body guaranteeing an easy and quick fitting and preserving comfort is still required in the literature [43]. Participant P3 represented an exception since the sensor’s data revealed limited frequency of use despite not experiencing challenges with donning. From the interview, we identified the cause for limited use in the health condition of the participant, for whom training was described as extremely exhausting.

Frequency of use of the device was also found to relate to the purpose of use of the exosuit. In the personalized questionnaire, participants P5 and P7 mentioned social and leisure activities (hiking and geocaching with the spouse and colleagues) and activities of daily living (going grocery, walking the dog, and going out with friends), respectively. The other participants reported in the personalized questionnaire to use the Myosuit exclusively as a training device. Participant P1 reported social activities in the questionnaire but explicitly mentioned during the interview to consider walking with the spouse as a training and not as a leisure activity.

. Therefore, from the collated results of qualitative and quantitative analysis, we concluded that integrating the device in the user’s daily routine, is another determining factor for extensive use of exosuits.

### Exosuits should become a tool to increase motivation and adherence to training without the supervision of a therapist

During the interview, four participants (P1, P2, P6 and P7) reported to train on average without the Myosuit, at a physiotherapist center, 45 min, once per week. P5 mentioned to perform weight training without the Myosuit, at a physiotherapist center, 45 min, twice per week. P4 did not train without the Myosuit. Overall, without the Myosuit, none of the participants reached the 150–300 min of moderate-intensity aerobic physical activity throughout the week that WHO suggests for adults living with disability [4]. All participants but one used the Myosuit as an adjunct to conventional physiotherapy, highlighting the potential of an exosuit to increase the dose of training and to push the users to be more physically active. However, only two participants (P5 and P7) exceeded the WHO recommendation when the duration of use of the Myosuit per week was also taken into account. The finding that participants do not often train without the Myosuit make us believe that lack of motivation to train was an additional factor that restrained participants from exercising frequently. It was expressed by the participants as limited time availability or everyday life’s exhaustion. Therefore, users should be encouraged to exercise more, and exosuits could be better exploited in this direction. Indeed, the sensors embedded in the suit could be used to provide real-time feedbacks on the activity level and quality of movement. Real-time feedback from continuous self-monitoring from wearable technology is expected to be useful to enhance lifestyle changes and improve the adoption of these devices [44]. Furthermore, we strongly believe that physiotherapists could play a fundamental role in increasing the frequency of use of technologies at home. We foresee the possibility of using an exosuit to replicate the training learnt from the physiotherapist while being at home. For this to happen, therapists should be willing to incorporate the technology in their sessions to instruct patients on how to properly handle the device, which exercise to perform and how often and how long to practice. However, participants in our study highlighted physiotherapists’ current tendency to favor conventional therapy rather than attempting robot-assisted training.

### Limitations and further work

Our study included a limited sample size. However, being the Myosuit one of the very few exosuits that private users have at their disposal at home, the total number of users with community-based experience of any exosuit is by itself extremely limited. Selection bias towards participants with high interest in the technology might have influenced the outcome of the study. Furthermore, we included participants with diverse demographics. However, the external validity is higher in a case series that includes a diverse range of participants because the study sample is more likely to be exemplary of the population of interest [45]. In these regards, we believe that the cohort of users that participated in our study is highly representative of the population that might be willing to purchase and use an exosuit at home and in the community. All participants lived in small villages or municipality in Switzerland and had the disease for at least 6 years, which might have influenced the perception of the disease in the public and social interaction in the community. To reduce recall bias, that might have influenced participants’ answers, most closed- and open-ended questions were formulated in the present and we avoid comparisons with past activities. Furthermore, activity level was directly extracted from the sensors embedded in the device and used to ensure answers’ trustworthiness.

Although participants perceived numerous benefits related to the use of the Myosuit, we were not able, within this study, to disclose how the adoption of the device in daily life influenced the quality of life of participants and improved their secondary health condition. In fact, being an exploratory design, our study did not allow us to draw structured conclusions on the impact of the exosuit on mobility and quality-of-life of users when used outside a clinic and outside the context of a controlled study protocol. Future studies should investigate whether using an exosuitregularly at home and in the community increases life satisfaction, quality-of-life and general health of users. However, we strongly believe in the importance of assessing user perspective using qualitative research. Indeed, although laboratory tests provide more structured results, the high level of control and supervision does not allow to capture factors that influence usability in the real world [46].

## Conclusion

In the current study we showed good usability of an exosuit. Users were satisfied with its low weight, reduced dimensions, safety, and comfort. We demonstrated the possibility of exploiting the Myosuit in various scenarios outside the laboratory and clinical settings and the participants mainly used the device for exercising at home and outside. The frequency of use was limited compared to the recommendations from the World Health Organization, suggesting that daily life context of use poses further challenges that should be considered to ensure increased acceptance of newly developed exosuits in unsupervised environments. Primarily, the design of exosuits should focus on meeting the users' claim for independent use and increased adjustability. The ability to don and operate the device independently is an extremely important factor for increasing the frequency of use of such technologies at home and in the community. However, we speculated that technical limitations of the Myosuit were not the only factors preventing extensive use. Societal aspects and lack of motivation were found to also limit the frequency of use. In this direction, an exosuit could be used to provide real-time feedback on the activity level and quality of performed movement. Real-time feedback from continuous self-monitoring is expected to play a motivational role in increasing technology adoption. Finally, our study highlights the importance of conducting such qualitative research to include users’ perspectives in the developmental process of these technologies and point out the challenges related to a specific context of use. We believe these results have greater generalizability to real-life contexts than those collected in laboratory settings.

## Supplementary Information

**Additional file 1: Figure S1. **Participants’ level of agreement with statements regarding device fitting, independent use, learnability, ease of control and acceptance within the community. **Table S1.** Personalized questionnaire. **Table S2.** Guidelines for the open-ended interview. **Table S3.** Participants’ exemplary statements from the interview transcripts.

## Data Availability

Data and materials can be made available upon request to the authors.

## Abbreviations

WHO: World Health Organization
SCI: Spinal cord injury
QUEST: Quebec User Evaluation of Satisfaction with assistive Technology
SUS: System usability survey
LEFS: Lower extremity functional scale
IMU: Inertial measurement unit
PT: PhysioTherapist
ADL: Activity of daily living

## References

[1] Beckerman H, De Groot V, Scholten MA, Kempen JCE, Lankhorst GJ (2010). Physical activity behavior of people with multiple sclerosis: Understanding how they can become more physically active. Phys Ther.

[2] Ginis KAM (2011). The development of evidence-informed physical activity guidelines for adults with spinal cord injury. Spinal Cord.

[3] Fini NA, Holland AE, Keating J, Simek J, Bernhardt J (2017). How physically active are people following stroke? Systematic review and quantitative synthesis. Phys Ther.

[4] “WHO recommendations on physical activity.” https://www.who.int/news-room/fact-sheets/detail/physical-activity. Accessed 19 Apr 2022.

[5] Rimmer JH, Schiller W, De Chen M (2012). Effects of disability-associated low energy expenditure deconditioning syndrome. Exerc Sport Sci Rev.

[6] Noreau L, Shephard RJ (1995). Spinal cord injury, exercise and quality of life. Sport Med.

[7] Jain NB, Sullivan M, Kazis LE, Tun CG, Garshick E (2007). Factors associated with health-related quality of life in chronic spinal cord injury. Am J Phys Med Rehabil.

[8] Gillis A, MacDonald B (2005). Deconditioning in the hospitalized elderly. Can Nurse.

[9] Arun Jayaraman PT, Rymer WZ (2017). Exoskeletons for rehabilitation and personal mobility: Creating clinical evidence. Biosyst Biorob.

[10] Lee G (2017). Reducing the metabolic cost of running with a tethered soft exosuit. Sci Robot.

[11] Galle S, Malcolm P, Collins SH, De Clercq D (2017). Reducing the metabolic cost of walking with an ankle exoskeleton: interaction between actuation timing and power. J Neuroeng Rehabil..

[12] Malcolm P, Derave W, Galle S, De Clercq D (2013). A simple exoskeleton that assists plantarflexion can reduce the metabolic cost of human walking. PLoS ONE.

[13] Mooney LM, Rouse EJ, Herr HM (2014). Autonomous exoskeleton reduces metabolic cost of human walking. J Neuroeng Rehabil..

[14] Kim J (2019). Reducing the metabolic rate of walking and running with a versatile, portable exosuit. Science (80-).

[15] Haufe FL, Wolf P, Duarte JE, Riener R, Xiloyannis M. Increasing exercise intensity during outside walking training with a wearable robot. In: 2020 8th IEEE RAS/EMBS International Conference for Biomedical Robotics and Biomechatronics (BioRob), 2020, pp. 390–395. 10.1109/BioRob49111.2020.9224408.

[16] Benson I, Hart K, Tussler D, Van Middendorp JJ (2016). Lower-limb exoskeletons for individuals with chronic spinal cord injury: findings from a feasibility study. Clin Rehabil.

[17] Nilsson A, Vreede KS, Häglund V, Kawamoto H, Sankai Y, Borg J (2014). Gait training early after stroke with a new exoskeleton—the hybrid assistive limb: a study of safety and feasibility. J Neuroeng Rehabil..

[18] Buesing C (2015). Effects of a wearable exoskeleton stride management assist system (SMA®) on spatiotemporal gait characteristics in individuals after stroke: a randomized controlled trial. J Neuroeng Rehabil.

[19] Haufe FL, Schmidt K, Duarte JE, Wolf P, Riener R, Xiloyannis M (2020). Activity-based training with the Myosuit: a safety and feasibility study across diverse gait disorders. J Neuroeng Rehabil.

[20] Meyer JT, Gassert R, Lambercy O (2021). An analysis of usability evaluation practices and contexts of use in wearable robotics. J Neuroeng Rehabil.

[21] Manns PJ, Hurd C, Yang JF (2019). Perspectives of people with spinal cord injury learning to walk using a powered exoskeleton. J Neuroeng Rehabil.

[22] Poritz JMP, Taylor HB, Francisco G, Chang SH (2020). User satisfaction with lower limb wearable robotic exoskeletons. Disabil Rehabil Assist Technol.

[23] Vaughan-Graham J, Brooks D, Rose L, Nejat G, Pons J, Patterson K (2020). Exoskeleton use in post-stroke gait rehabilitation: a qualitative study of the perspectives of persons post-stroke and physiotherapists. J Neuroeng Rehabil.

[24] Lajeunesse V, Routhier F, Vincent C, Lettre J, Michaud F (2018). Perspectives of individuals with incomplete spinal cord injury concerning the usability of lower limb exoskeletons: an exploratory study. Technol Disabil.

[25] Dobkin BH, Duncan PW (2012). Should body weight-supported treadmill training and robotic-assistive steppers for locomotor training trot back to the starting gate?. Neurorehabil Neural Repair.

[26] Hill D, Holloway CS, Morgado Ramirez DZ, Smitham P, Pappas Y (2017). What are user perspectives of exoskeleton technology? A literature review. Int J Technol Assess Health Care.

[27] van Dijsseldonk RB, van Nes IJW, Geurts ACH, Keijsers NLW (2020). Exoskeleton home and community use in people with complete spinal cord injury. Sci Rep.

[28] Singal AG, Higgins PDR, Waljee AK (2014). A primer on effectiveness and efficacy trials. Clin Transl Gastroenterol.

[29] Binkley JM, Lott SA (1999). The lower extremity functional scale (LEFS): scale development, measurement properties, and clinical application. Phys Ther.

[30] Schmidt K (2017). The myosuit: Bi-articular anti-gravity exosuit that reduces hip extensor activity in sitting transfers. Front Neurorobot.

[31] “Myoswiss – Healthcare Professionals.” https://myo.swiss/en/resources-healthcare-professionals/. Accessed 23 Nov 2021.

[32] Brooke J (1996). SUS: a ‘quick and dirty’ usability scale. Usability Eval Ind.

[33] Wolff J, Parker C, Borisoff J, Ben Mortenson W, Mattie J (2014). A survey of stakeholder perspectives on exoskeleton technology. J Neuroeng Rehabil.

[34] Louie DR, Mortenson WB, Durocher M, Teasell R, Yao J, Eng JJ (2020). Exoskeleton for post-stroke recovery of ambulation (ExStRA): Study protocol for a mixed-methods study investigating the efficacy and acceptance of an exoskeleton-based physical therapy program during stroke inpatient rehabilitation. BMC Neurol.

[35] Kinnett-Hopkins D (2020). Users with spinal cord injury experience of robotic Locomotor exoskeletons: a qualitative study of the benefits, limitations, and recommendations. J Neuroeng Rehabil.

[36] Tong A, Sainsbury P, Craig J (2007). Consolidated criteria for reporting qualitative research (COREQ): a 32-item checklist for interviews and focus groups. Int J Qual Heal Care.

[37] Thomas DR (2006). A general inductive approach for analyzing qualitative evaluation data. Am J Eval.

[38] Bangor A, Staff T, Kortum P, Miller J, Staff T (2009). Determining what individual SUS scores mean: adding an adjective rating scale. J usability Stud.

[39] Bangor A, Kortum PT, Miller JT (2008). An empirical evaluation of the system usability scale. Int J Hum Comput Interact.

[40] van Dijsseldonk RB, Vriezekolk JE, Keijsers NLW, Geurts ACH, van Nes IJW (2022). Needs and wishes for the future lower limb exoskeleton: an interview study among people with spinal cord injury with community-based exoskeleton experience. Disabil Rehabil.

[41] Wright A (2020). Effect of combined home-based, overground robotic-assisted gait training and usual physiotherapy on clinical functional outcomes in people with chronic stroke: a randomized controlled trial. Clin Rehabil.

[42] Bryce TN, Dijkers MP, Kozlowski AJ (2015). Framework for assessment of the usability of lower-extremity robotic exoskeletal orthoses. Am J Phys Med Rehabil.

[43] Xiloyannis M (2021). Soft robotic suits: state of the art, core technologies, and open challenges. IEEE Trans Robot.

[44] Brennan L, Zubiete ED, Caulfield B (2020). Feedback design in targeted exercise digital biofeedback systems for home rehabilitation: a scoping review. Sensors (Switzerland).

[45] Kooistra B, Dijkman B, Einhorn TA, Bhandari M (2009). How to design a good case series. J Bone Jt Surg.

[46] Maguire M (2001). Context of use within usability activities. Int J Hum Comput Stud.

