# Supplementary material for: Usability of an exosuit in domestic and community environments
Source: J Neuroeng Rehabil. 2022 Dec 1;19:131. doi: 10.1186/s12984-022-01103-6 (PMC9714034; doi:10.1186/s12984-022-01103-6)
Supplement: Supplementary file 1 — Additional file 1: Figure S1. Participants’ level of agreement with statements regarding device fitting, independent use, learnability, ease of control and acceptance within the community. Table S1. Personalized questionnaire. Table S2. Guidelines for the open-ended interview. Table S3. Participants’ exemplary statements from the interview transcripts. [file 12984_2022_1103_MOESM1_ESM.docx]

# **Additional file 1**


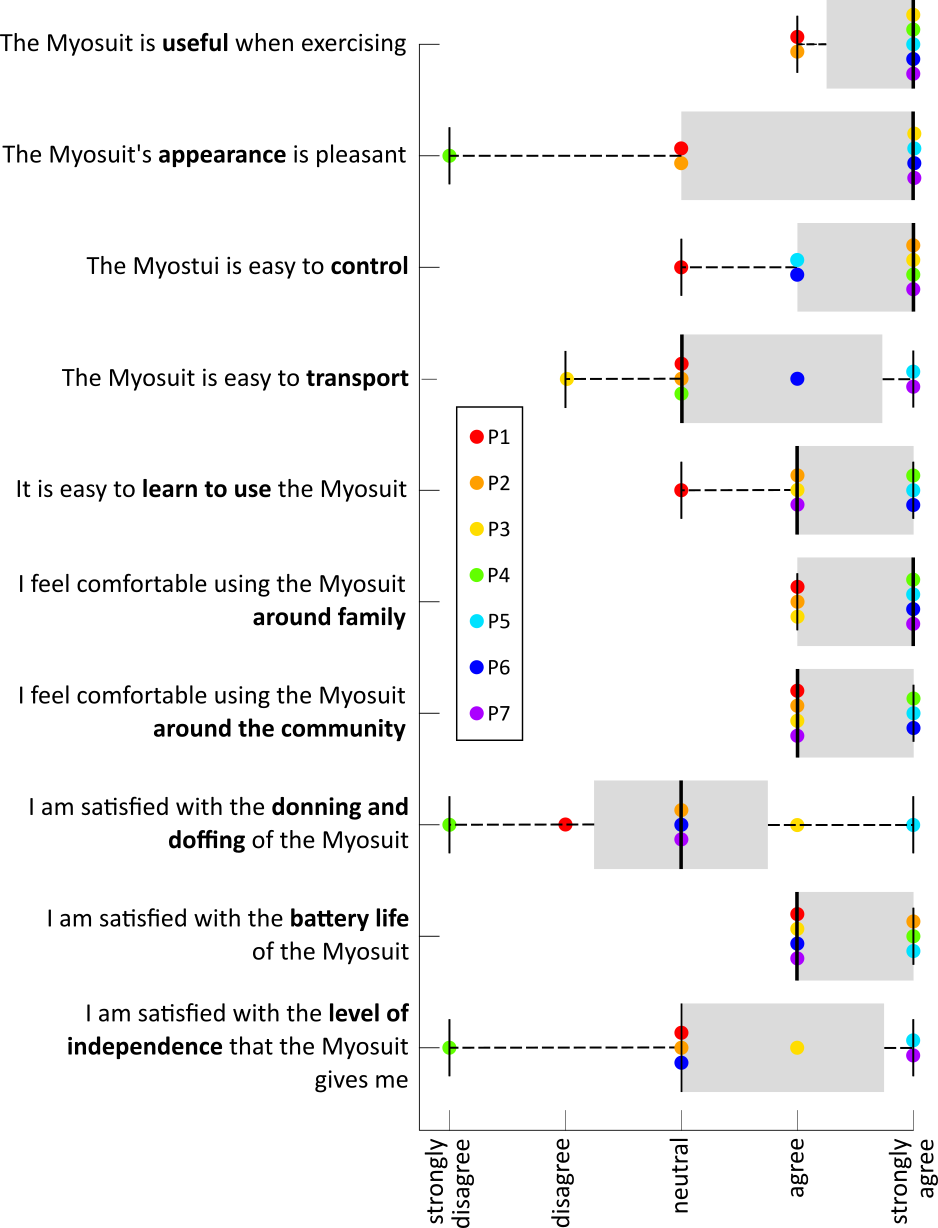


**Figure S1. Participants’ level of agreement with statements regarding device fitting, independent use, learnability, ease of control and acceptance within the community.** Within the personalized questionnaire, the participants were asked to rate their level of agreements with 10 statements regarding their experience with the exosuit. A five-points Likert scale was used from strongly disagree to strongly agree. The thick black line indicates the median, and the bottom and top edges of the box indicate the 25th and 75th percentiles, respectively. The whiskers extend to the most extreme data points not considered outliers. Each color represents one participant.

**Table S1. Personalized questionnaire.**

| The following questionnaire is intended to understand your experience with the Myosuit. Please answer all questions as honestly as possible.  For each statement, please select one of the 5 possible answers.  Do you use the Myosuit….   \|  \| Never \| Rarely \| Sometimes \| Often \| Always \| \| --- \| --- \| --- \| --- \| --- \| --- \| \| at home? \| 1 \| 2 \| 3 \| 4 \| 5 \| \| at work? \| 1 \| 2 \| 3 \| 4 \| 5 \| \| at the fitness centre / gym? \| 1 \| 2 \| 3 \| 4 \| 5 \| \| at the physiotherapist centre? \| 1 \| 2 \| 3 \| 4 \| 5 \| \| outdoors (e.g. nature, neighbourhood)? \| 1 \| 2 \| 3 \| 4 \| 5 \| \| other places: \| 1 \| 2 \| 3 \| 4 \| 5 \| \| Please write examples: \|  \|  \|  \|  \|  \|   Do you use the Myosuit for….   \|  \| Never \| Rarely \| Sometimes \| Often \| Always \| \| --- \| --- \| --- \| --- \| --- \| --- \| \| exercising without the physiotherapist? \| 1 \| 2 \| 3 \| 4 \| 5 \| \| exercising with the physiotherapist in presence? \| 1 \| 2 \| 3 \| 4 \| 5 \| \| exercising with the physiotherapist remotely? \| 1 \| 2 \| 3 \| 4 \| 5 \| \| participating in social activities? \| 1 \| 2 \| 3 \| 4 \| 5 \| \| Please write examples: \|  \|  \|  \|  \|  \| \| daily life activities? \| 1 \| 2 \| 3 \| 4 \| 5 \| \| Please write examples: \|  \|  \|  \|  \|  \| \| other activities? \| 1 \| 2 \| 3 \| 4 \| 5 \| \| Please write examples: \|  \|  \|  \|  \|  \|   Please rate the following statements.   \|  \| Strongly disagree \| Disagree \| Neutral \| Agree \| Strongly agree \| \| --- \| --- \| --- \| --- \| --- \| --- \| \| The Myosuit is useful when exercising. \| 1 \| 2 \| 3 \| 4 \| 5 \| \| The Myosuit's appearance is pleasant. \| 1 \| 2 \| 3 \| 4 \| 5 \| \| The Myosuit is easy to control. \| 1 \| 2 \| 3 \| 4 \| 5 \| \| The Myosuit is easy to transport. \| 1 \| 2 \| 3 \| 4 \| 5 \| \| It is easy to learn to use the Myosuit. \| 1 \| 2 \| 3 \| 4 \| 5 \| \| I feel comfortable using the Myosuit around family. \| 1 \| 2 \| 3 \| 4 \| 5 \| \| I feel comfortable using the Myosuit around the community. \| 1 \| 2 \| 3 \| 4 \| 5 \| \| I am satisfied with the donning and doffing of the Myosuit. \| 1 \| 2 \| 3 \| 4 \| 5 \| \| I am satisfied with the battery life of the Myosuit. \| 1 \| 2 \| 3 \| 4 \| 5 \| \| I am satisfied with the level of independence that the Myosuit gives me. \| 1 \| 2 \| 3 \| 4 \| 5 \|   **Demographic data**  First name _____________________________  Family name _____________________________  Gender _____________________________  Age _____________________________  Place of residence _____________________________  Number of people  in your household _____________________________  (including yourself)   \|  \| 1^st^ pathology \| 2^nd^ pathology (optional) \| \| --- \| --- \| --- \| \| Pathology \| ___________________________ \| _______________________________ \| \| Time since first diagnosis (yyyy) \| ___________________________ \| _______________________________ \| |
| --- | --- | --- | --- | --- | --- | --- | --- | --- | --- | --- | --- | --- | --- | --- | --- | --- | --- | --- | --- | --- | --- | --- | --- | --- | --- | --- | --- | --- | --- | --- | --- | --- | --- | --- | --- | --- | --- | --- | --- | --- | --- | --- | --- | --- | --- | --- | --- | --- | --- | --- | --- | --- | --- | --- | --- | --- | --- | --- | --- | --- | --- | --- | --- | --- | --- | --- | --- | --- | --- | --- | --- | --- | --- | --- | --- | --- | --- | --- | --- | --- | --- | --- | --- | --- | --- | --- | --- | --- | --- | --- | --- | --- | --- | --- | --- | --- | --- | --- | --- | --- | --- | --- | --- | --- | --- | --- | --- | --- | --- | --- | --- | --- | --- | --- | --- | --- | --- | --- | --- | --- | --- | --- | --- | --- | --- | --- | --- | --- | --- | --- | --- | --- | --- | --- | --- | --- | --- | --- | --- | --- | --- | --- | --- | --- | --- | --- | --- | --- | --- | --- | --- | --- | --- | --- | --- | --- | --- | --- | --- | --- | --- | --- | --- | --- | --- | --- | --- | --- | --- | --- | --- | --- | --- | --- | --- | --- | --- | --- | --- | --- | --- | --- | --- |

**Table S2. Guidelines for the open-ended interview.** Based on this guideline, the investigator asked specific questions to each participant. The investigator analysed the questionnaires’ answers before the interview to get a first idea of the participant’s impressions but never used explicit references to the questionnaires’ answers or gave no information for known during the interview. The investigator took additional notes for each participant before the interview to guide the discussion.

| Today I would like to discuss with you about your personal experience with the Myosuit.  Please answer all the questions as honestly as possible.  Please remember that the company won’t be informed of any of your answers.  The interview will be audio recorded.  If anything is unclear or you have any questions, please stop me at any time.   - Can you please introduce yourself and tell me why you bought the Myosuit? |
| --- |
| - Why do you continue using the Myosuit? |
| - What features of the Myosuit do you like? What features don’t you like? |
| - What activities do you perform with the Myosuit? |
| - What are the advantages of having the Myosuit at home? What are the disadvantages? |
| - What would you like to change to use it more frequently at home? |

**Table S3. Participants’ exemplary statements from the interview transcripts.**

| **Usability** | | |
| --- | --- | --- |
| Technical features | Exo attractors | “It is comfortable to wear, even for people with fewer muscles, it doesn't hurt you. The backpack is not perceived as disturbing weight. Once you have it on and turn the Myosuit on, that's not anymore. So, from the outside, it may seem that it's way too heavy, but this is not the case at all.” (P2) |
|  |  | “It is very compact with the backpack. It's great that it's not a whole suit.” (P4) |
|  |  | “It's actually very light. […] There [operating the device] I'm mostly independent. I can make the settings alone.” (P7) |
|  | Exo detractors | “[The straps of the Myosuit] tie you up pretty much when you have to sit somewhere longer.” (P1) |
|  |  | “It is not very comfortable when you have the backpack on and then you sit down somewhere and lean on.” (P3) |
|  |  | “I don't really like Velcro fasteners for myself personally. They get trapped everywhere, right?” (P4) |
| Factors limiting use | Donning | “It's hard for me to be able to use the Myosuit on a regular basis. Because I always depend on someone. And that's frustrating in a certain way. If I put on the Myosuit on myself and don't need someone for it, then I would go walking [alone] with the walker too.” (P1) |
|  |  | “At the moment, I think twice about [exercising with the Myosuit]; "do I do it now and get exhausted with it [the donning] or do I wait until someone comes to support me?". But then when that person is there, there are usually other necessities.” (P6) |
|  | Lack of motivation | “I'm still working 100%. For me, normal everyday life is already extremely exhausting. It's practically a matter of time, too” (P4) |
|  | Preserved body functions | “At home in the house I don't use it. I just try the stuff I can still do without [the Myosuit]. My legs are still enough at home.” (P5) |
| User suggestions | Wish for changes | “The ideal device would be normal pants with the technology inside.” (P2) |
|  |  | “What I'm imagining is really like the Tony Stark, the nano suit. One on the front, one on the back and zack. So that would be great.” (P4) |
| **Personal experience** | | |
| Goal setting | Activity augmentation | “The goal is for me to walk longer. So, for example, I could go further with my wife.” (P2) |
|  |  | “My goal is to walk longer distances so I can go hiking in the mountains again.” (P5) |
|  | Performance improvement | “The goal is simply to improve the gait pattern so that I walk easier and better.” (P3) |
|  |  | “The goal with the Myosuit is that I actually have a really good gait pattern. Because then I can walk normally and I'm sure I stumble less or have more endurance. That's the goal.” (P4) |
|  | Delay disease progression | “I want to try to keep this walking ability, but also the strength in the legs. Because I'm going worse and worse all the time and if this "worsening" is slowed down, then I'm benefiting from it.” (P6) |
| Perceived benefits | Physical benefits (restorative) | “I was almost not able to carry a cup of tea from the kitchen to the dining table at first. I spilled the tea because I wasn't stable enough from the pelvis and even jerked in the movements. And now I can transfer a cup of tea on my own [without the Myosuit], simply because the stability of the pelvis improved.” (P1) |
|  |  | “It prevents me from getting pain in joints that are loaded incorrectly or also muscles.” (P3) |
|  |  | “My body is getting used to remembering the normal walking sequences, just like pelvis with legs and so on. That's coming back for about two days.” (P5) |
|  |  | “After the 1.8 km with the Myosuit, I felt "healthy" exertion and had a "healthy" fatigue. I don't even know how to say it any other way.... I can only describe it as "healthy" or otherwise I always feel "sick tired" after everything. And with the Myosuit, I perceived a healthy fatigue.” (P6) |
|  | Physical benefits (assistive) | “Normally, I can walk outside, maybe a maximum of 20 minutes. But also with walking sticks, so I have walking sticks like that, and with the Myosuit I really get an hour to an hour and a half, a break in between, to two hours.” (P5) |
|  |  | “I sometimes block out my surroundings because I'm just concentrated on walking. And with the Myosuit is usually not as strong as without.” (P6) |
|  |  | “[Without the Myosuit] I am very much slower when I move. With the Myosuit I'm always quicker at the destination.” (P7) |
|  | Psycho-social benefits | “When I'm in a wheelchair and I am driven, I feel like a disabled person, as opposed to wearing the Myosuit. You are active and perceived as active, much less than disabled. Someone in a wheelchair, that's more negative, right?” (P1) |
|  |  | “I did not notice improvements within the society. I have such a good environment.” (P2) |
|  |  | “So actually, I really had adrenaline spurts like that, or endorphin spurts, because you can just walk normally again. It's really indescribable.” (P4) |
|  |  | “Social is going again in the wood with my husband and moving outside with others clearly.” (P5) |
| **Context of use** | | |
| Exo at home | Integration in daily routine | “You can individually incorporate it into your day. It has the advantage that I can train more often then, not just once a week. You can use Myosuit when it's convenient.” (P3) |
|  |  | “I don't think it's an advantage now that I have it at home. Because if I don't automatically have someone to help me. It takes a lot more organization as if I have a fixed appointment.” (P1) |
|  | Learning to use the device | “I wouldn't recommend anyone right now to just buy the Myosuit, put it on and just walk with it. In my opinion, you can only really see the benefits of the Myosuit after the fourth training with the physiotherapist.” (P1) |
|  |  | “I asked for an extra session because the official introduction wasn't enough for me. Because I wanted to get to know the device properly. If you have enough time and you can go through the process several times, then it's actually easy afterwards.” (P7) |
| Exo at PT center | PT feedbacks | “I appreciate doing it with therapeutic support because I have to concentrate so much on the movements, that I often don't notice if I'm moving in the wrong way. And with a therapist, they can correct you. Which is also motivating, of course. If no one is there, you give up, if someone motivates you, then you just keep going for two or three more [repetitions].” (P6) |
|  | Set-up concerns | “He didn’t know what exercises he was supposed to do with me because he doesn't have the experience with devices like that, so he asked me to train without it.” (P2) |
|  |  | “My physiotherapist is not set up for this.” (P5) |
| Exo in the community | Public reaction | “Then of course everyone comes and wants to know how it works. You can't go anywhere without being addressed. And that takes longer. It's also really tedious for my family.” (P4) |
|  |  | “There are a lot of brave people who ask why I wear this and how it helps me. And I am happy to discuss because I noticed that many people know someone who can't walk so well and extremely many with multiple sclerosis. Because it helps me, maybe it can help someone else too.” (P5) |
|  | Being noticeable | “When walking around, then of course one is a certain eye-catcher, in a way. But that's totally fine for me. Because also with my wheelchair, even when I'm walking, I am always visible.” (P6) |
|  |  | “[Being noticeable in the community] doesn't bother me personally. I came across the Myosuit after 21, 22 years. My head is a lot freer for stuff like that.” (P7) |
